# A Simple Protocol for Isolating Hemolymph from Single *Drosophila melanogaster* Adult Flies

**DOI:** 10.3390/mps6050100

**Published:** 2023-10-12

**Authors:** Kirah Jones, Ioannis Eleftherianos

**Affiliations:** Department of Biological Sciences, The George Washington University, Washington, DC 20052, USA

**Keywords:** *Drosophila*, infection, innate immunity, hemolymph, humoral response, cellular response

## Abstract

*Drosophila melanogaster* is an excellent model for dissecting innate immune signaling and functions. Humoral and cellular immune mechanisms in the fly take place in the hemolymph, where host defense components are secreted and act in response to microbial invaders. Studying hemolymph factors is critical for understanding the regulation of the host’s antimicrobial immune system. Therefore, methods for extracting the fly hemolymph efficiently and in sufficient quantities are essential for isolating and characterizing immune proteins and peptides. Here, we describe a novel and simple hemolymph isolation protocol for single *D. melanogaster* male and female adults. This procedure substantially improves the already used technique and allows fly immunologists to explore innate immune hemolymph activity in *D. melanogaster* individuals.

## 1. Introduction

Insects are great models for analyzing the molecular and mechanistic bases of fundamental biological processes. In particular, the fruit fly *Drosophila melanogaster* is extremely useful in biomedical research because of its extensive genetic toolkit, including evolutionary-conserved signaling pathways, easiness for culturing and maintenance in the lab, and experimental amenability to controlled functional studies at both organismal and tissue-specific levels [1,2].

*D. melanogaster* represents an ideal animal host model to address novel questions in the innate antimicrobial immune response [3,4]. The *D. melanogaster* immunology field is most famous for the aspects of its immunity that are shared with humans and other mammals [5]. These include the signaling pathways employed for microbial detection, the production of antimicrobial peptides, and the cellular immune activity of hemocytes (equivalent to mammalian blood cells) [6]. Most of these immune processes take place in the hemolymph (equivalent to mammalian blood), which fills the cavity of the fly’s open circulatory system in the body and employs several secreted proteins and peptides that suppress pathogen replication [7].

A detailed analysis of hemolymph immune factors requires the use of a robust method that can isolate sufficient amounts of hemolymph from *D. melanogaster* adult flies [8]. However, due to the variation in immune capacity between individual flies, it is imperative to be able to perform functional assays in single flies [9,10]. Here, we describe a simple protocol for isolating hemolymph from individual male and female *D. melanogaster* adult flies. Developing a hemocyte extraction technique in an animal model with evolutionary conserved molecular and physiological features could potentially reveal novel insights into the function of host hemolymph components and hemocyte mechanisms that combat pathogenic infections.

## 2. Experimental Design

This protocol for hemolymph extraction parallels the techniques and materials that are routinely used to inject *D. melanogaster*, so it does not require an additional learning curve or materials if there is familiarity when working with this model organism. It requires a standard nanoinjector and capillary needle system with slight modifications to reverse the process and achieve nano-extraction. For hemocyte collection, adult flies are first injected with a protease inhibitor (PI), and then the directional flow of the nanoinjector is reversed to withdraw the hemolymph backward up into the capillary. After sufficient hemolymphs have been collected, the contents of the capillary needle are emptied into another vial containing additional PI to await further analysis.

The sequence and timing of injecting PI into flies and withdrawing hemolymph varies depending on the intent of comparing hemocyte activity between individuals or experimental groups with the purpose of preserving the viability of cells and avoiding cross-contamination. The ability to include certain controls with this technique also varies between individuals and groups and the programming settings of the nanoinjector.

Previous hemolymph extraction methods in *D. melanogaster* have difficulty quantifying the amount of hemolymph extracted from each individual fly when spinning down a collective group [11]. This limitation is still present when using a single needle to collect the hemolymph from multiple individuals, and the nanoinjector is unable to be programmed to withdraw a precise volume. However, when extracting the hemolymph from individual flies, the precise volume delivered by the nanoinjector can be used as a control for a consistent volume of hemolymph extracted from each fly. By using a fresh needle filled to its capacity with PI, the difference in 100 nL that is displaced from the capillary and into the fly becomes the maximum volume that can be retrieved.

### 2.1. Materials

*D. melanogaster*: The Oregon-R line was maintained at 25 °C with a 12 h light cycle. The flies were kept on a diet that consisted of soy-based cornmeal (LabExpress, Ann Arbor, MI, USA) supplemented with yeast. All experiments were carried out with male and female adult flies aged for approximately 5–10 days.

Protease inhibitor Cocktail (Sigma-Aldrich, St. Louis, MO, USA, Cat. P2714-1BTL Lot #065M4144V).

Mineral Oil (Millipore Sigma, Burlington, MA, USA, Cat. 8042-47-5).

Sodium Phosphate Buffer (ThermoFisher Scientific, Waltham, MA, USA, Cat. 11-656-0205).

A total of 1.5 mL of microcentrifuge tubes were used (USA Scientific, Ocala, FL, USA, Cat. 1615-5510).

Glass Capillaries (Borosilicate capillary needles, Sutter Instruments, Novato, CA, USA, (Outside Diameter: 1.0 mm, Inside Diameter: 0.50 mm, Overall Length: 10 cm, Cat. BF100-50-10).

### 2.2. Equipment

Nanoject III Programable Nanoliter Injector (Drummond Scientific, Broomall, PA, USA).

Needle puller P-1000 (Sutter Instruments, Novato, CA, USA).

Nanodrop spectrophotometer (ThermoFisher Scientific, Waltham, MA, USA).

Stereomicroscope (Tritech Research, Inc., Los Angeles, CA, USA).

Centrifuge (Eppendorf, Framingham, MA, USA).

Carbon dioxide (CO_2_) anesthetizing system for *Drosophila* (Genesee Scientific, El Cajon, CA, USA).

### 2.3. Statistical Analysis

A comparison of hemocytes and protein collected from traditional centrifugation versus the nano-extraction method was repeated three times, each with two biological replicates, and the results were analyzed using an unpaired two-tailed *t*-test in GraphPad Prism 9. A comparison of hemocyte numbers, hemocyte viability, and protein concentrations collected from 10 individuals via the nano-extraction method and 40 individuals via centrifugation was repeated three times with biological replicates and two technical replicates per trial. The statistical analysis of data was performed using unpaired two-tailed *t*-test (hemocyte numbers), two-way ANOVA (hemocyte viability), and unpaired two-tailed *t*-test (protein concentrations) in GraphPad Prism 9. The average concentration of hemolymph collected from individual *D. melanogaster* male and female adult flies was repeated three times with 10 males and 10 females per trial, two technical replicates per sample, and the results were analyzed using unpaired two-tailed *t*-tests in GraphPad Prism 9.

## 3. Procedure

1. Prepare the nanoinjector per the manufacturer’s instructions and attach a glass capillary filled with mineral oil. Set the injector to “empty” to remove excess oil from the needle, and then fill the capillary needle with 5 μL of the diluted 2.5× protease inhibitor (PI) in 0.1 M of the sodium phosphate buffer (pH 7.4) per the manufacturer’s protocol. The flow rate is set at 69 nL/s and the fill rate at 30 nL/s.
2. Group Collection: Anesthetize the flies under carbon dioxide and inject individuals with 100 nL of PI. After all individuals are injected, set the nanoinjector to the “fill” setting.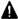 CRITICAL STEP TIP: Before withdrawing hemolymph from the flies, there should be PI left in the needle (approximately 10 nL) to come into contact with the extracted hemolymph and preserve it.
  - Reinsert the capillary into the mesothorax region of the adult fly and slightly pulse the nanoinjector for one to two seconds, creating a small vacuum allowing the hemolymph to freely draw upward into the needle.
  - Once hemolymph is extracted from all individuals and the contents of the needle are emptied into a 1.5 mL microcentrifuge tube containing 20 μL of 2.5× PI on ice.
  - OPTIONAL STEP: If extracting hemolymph from a large number of flies, periodically empty the collected hemolymph (approximately every 5–10 individuals) into the microcentrifuge tube on ice to preserve the hemolymph and repeat until all individuals are processed.
3. Individual Collection: While flies are anesthetized under CO_2_, prepare the nanoinjector similarly to 3.1 but fill the capillary with 2.5 μL of PI instead of 5 μL. Inject a single individual with 100 nL of PI and then immediately reverse the flow of the nanoinjector to collect the hemolymph.
  - Empty the contents of the needle into a 0.5 mL microcentrifuge tube containing 2.5 μL of 2.5× PI on ice.
  - Repeat this process for all individuals in the trial.
  - OPTIONAL STEP: You may change the needle in between individuals to avoid cross-contamination.
4. Gently vortex the tube with extracted hemolymph and store it on ice until the hemocytes are ready for analysis.
5. TROUBLESHOOTING: After creating the vacuum, you may need to slightly pull back on the needle in order for the hemolymph to flow backward into the needle.

## 4. Results and Discussion

**Figure 1:**
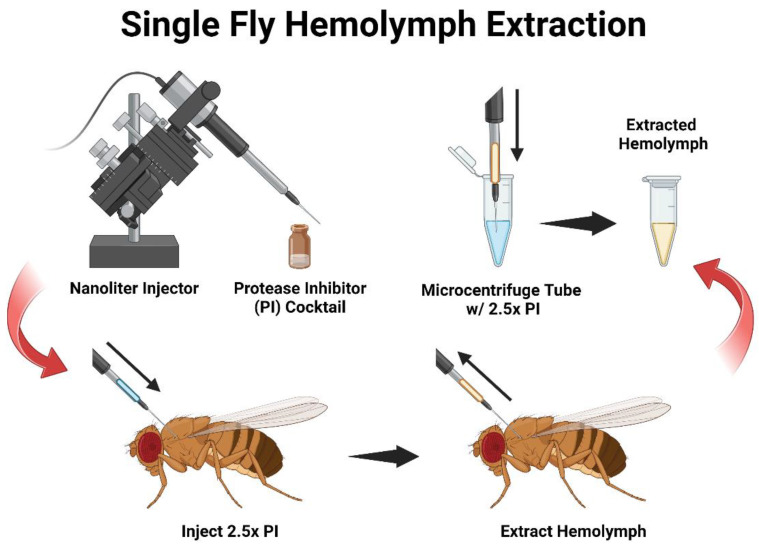
Diagram of the *D. melanogaster* hemolymph nano-extraction protocol. A schematic overview of the hemolymph nano-extraction collection method from *D. melanogaster* adult flies. The experimental setup includes a nanoliter injector prepared and filled with a protease inhibitor (PI) cocktail. The capillary needle is inserted into the mesothorax region of adult *D. melanogaster* to flush the main body cavity. Then, the flow of the nanoinjector is set to reverse to uptake hemolymph into the needle. Once the flow of hemolymph has been exhausted from an individual fly, the contents of the needle are emptied into a microcentrifuge tube with additional PI on ice for further analysis.

This protocol is accomplished with established equipment that is routinely used in *D. melanogaster* research. In contrast to a previously reported technique, our method employs a typical nanoinjector apparatus that is commonly used in several insect laboratories without requiring a device that generates airflow and pressure. Using this approach, we demonstrated that sufficient amounts of hemolymph can be collected from *D. melanogaster* adult female and male individuals [8]. A nanoliter injector is a critical tool for infection trials and can administer other immunoreactive compounds to survey their biological effects [12,13,14,15]. Our method uses this equipment with the dual purpose of delivering a designated volume and, in return, extracting hemolymph from individual adult flies. Here, we designed a hemolymph extraction setup of this protocol where a nanoliter injector is prepared with a glass capillary needle and filled with a protease inhibitor (PI) cocktail (Figure 1). The solution is then injected into an adult fly and subsequently retracted to withdraw hemolymph into the capillary. Then, the extracted hemolymph is emptied into a microcentrifuge tube to await further analysis. This nano-extraction protocol is easily applicable to those already skilled in fly nanoinjections and allows for the efficient collection of hemolymph from both groups and individual adult flies.

**Figure 2:**
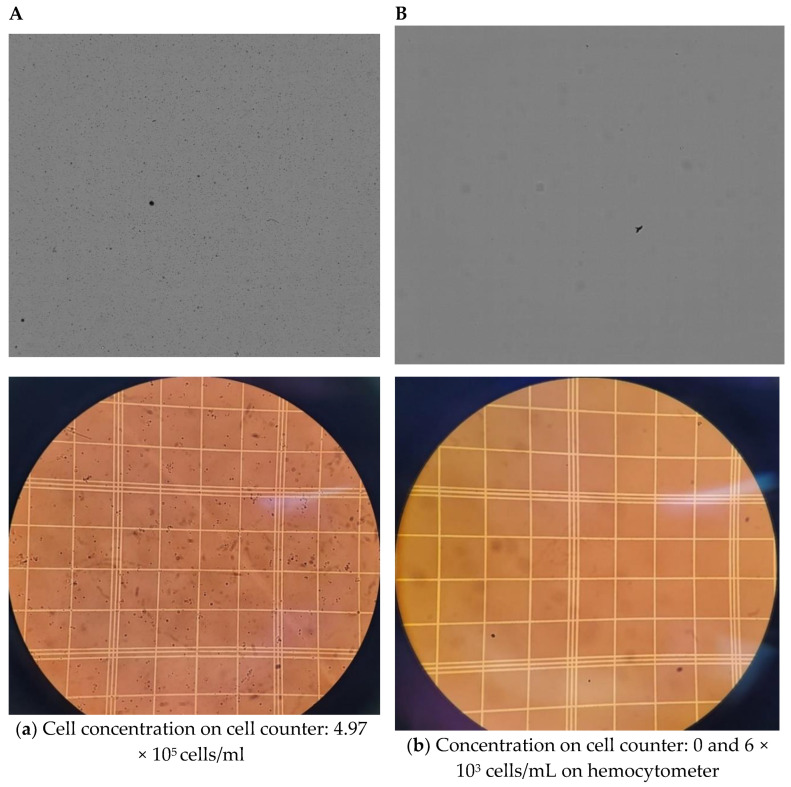

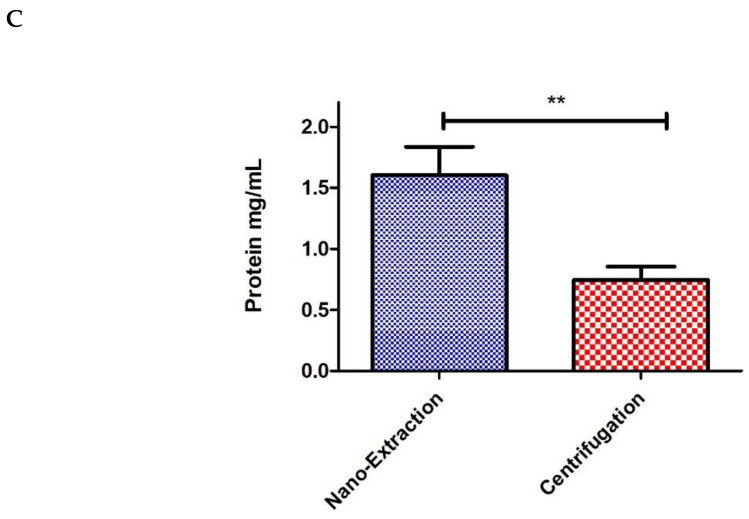
Comparison of hemocytes and protein collected from traditional centrifugation vs. nano-extraction. (**A**,**B**) Order of images: cell counter on top and hemocytometer below: (**A**) A group of 10 flies (5 males and 5 females) was injected with 100 nL of the protease inhibitor (PI), hemolymph was extracted with a nanoinjector and then emptied into 20 μL of PI. The sample was divided in half with 10 μL loaded into a cell counter and 10 μL into a hemocytometer. (**B**) A group of 10 flies (5 males and 5 females) was pricked with a sterile needle and placed in a 0.5 mL microcentrifuge tube with a hole punctured at the bottom tip with another sterile needle. Five 4 mm glass beads and 5 μL of PI were added on top of the flies and then stacked and spun down into a 1.5 mL microcentrifuge tube containing 20 μL of PI at 5000 rpm for five minutes. The sample was divided in half with 10 μL and loaded into a cell counter and 10 μL into a hemocytometer. (**C**) Hemolymph was extracted from two groups of 10 flies (5 males and 5 females) with two biological replicates following either method described in (**a**,**b**). Samples were loaded into a nanodrop spectrophotometer, and protein concentrations were quantified at A280 absorbance with two technical replicates; this process was repeated over 3 trials. Significant differences between the two hemocyte collection methods were calculated using an unpaired two-tailed *t*-test (** *p* = 0.0074, GraphPad Prism 9).

Initial experiments comparing nano-extraction versus the centrifugation method with an equal number of flies were proven to have limitations due to the cell counter machine (TC20, Bio-Rad, Hercules, CA, USA) being unable to quantify centrifuged samples containing hemolymph from only 10 flies. Images from the cell counter displayed minimal to no visible cells compared to its nano-extraction counterpart (Figure 2A,B). To confirm this disparity, cells were counted manually with a hemocytometer. The centrifuge method had cell concentrations in the 10^3^ range for samples containing 10 flies (Figure 2B), which is below the range of the machine’s capability. From these early findings, the nano-extraction method already displayed an advantage over centrifugation as it was capable of quantifying hemocytes consistently when working with a low number of individuals in a sample compared to centrifugation.

Next, we focused on the protein to see if the nano-extraction method was more efficient at collecting the protein compared to centrifugation while still working with a low number of individuals. The nano-extraction technique demonstrated a significant increase in protein concentration with a mean average that was double compared to the traditional centrifuge method (Figure 2C). From these observations, we can conclude that the nano-extraction method is a more efficient way of collecting hemolymph and generates a higher number of proteins and hemocytes than the previous method of centrifugation.

**Figure 3:**
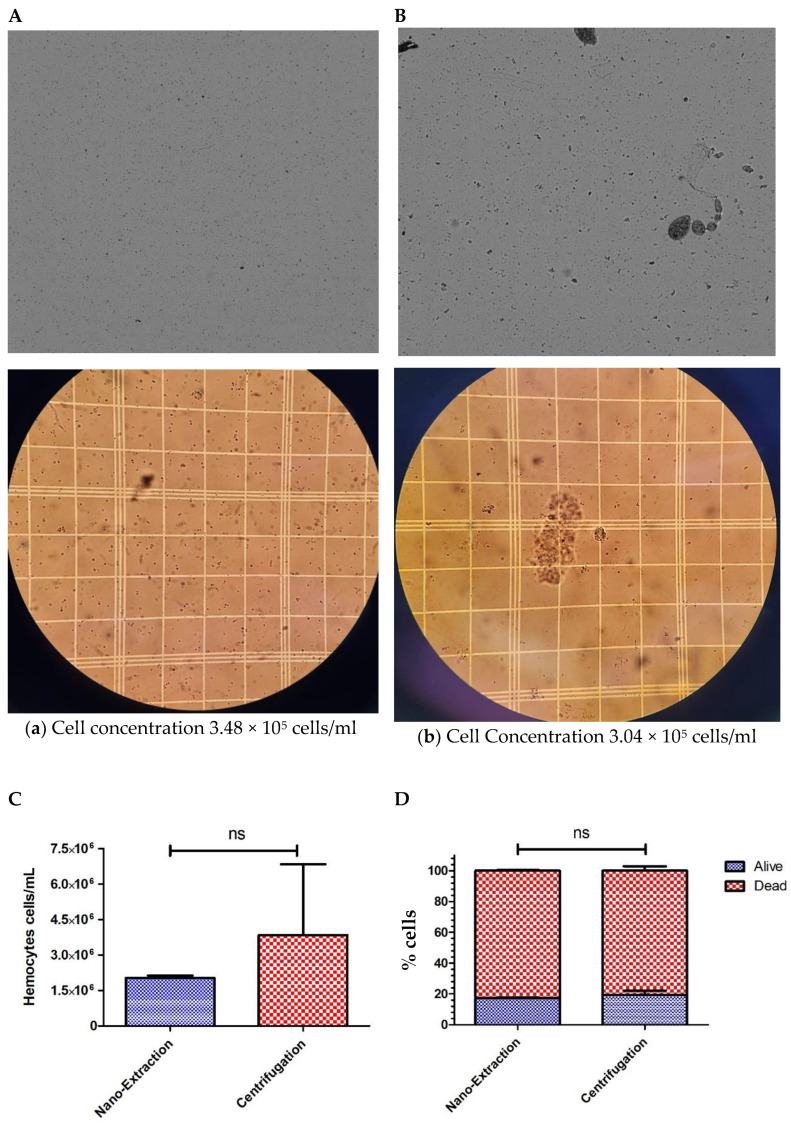

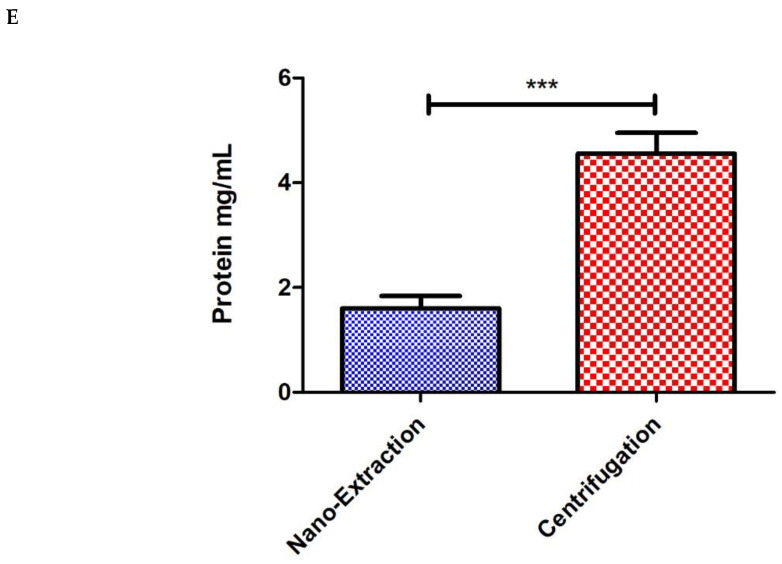
Comparison of hemocyte numbers, hemocyte viability, and protein concentrations collected from 10 individuals via the nano-extraction method and 40 individuals via centrifugation. (**A**,**B**) Order of images: cell counter on top and hemocytometer below: (**A**) A group of 10 flies (5 males and 5 females) were injected with 100 nL of the protease inhibitor (PI) hemolymph was extracted with a nanoinjector, and then emptied into 20 μL of PI. The sample was divided in half with 10 μL loaded into a cell counter and 10 μL into a hemocytometer; (**B**) A group of 40 flies (20 males and 20 females) was pricked with a sterile needle and then spun down into 20 μL of PI. The sample was divided in half with 10 μL and loaded into a cell counter and 10 μL into a hemocytometer. (**C**,**D**) Hemolymph was extracted from two groups following the methods described in (**a**,**b**) nano-extraction: 10 flies (5 males and 5 females), centrifugation: 40 flies (20 males and 20 females) with two biological replicates and two technical replicates with 4 readings per technical replicate. Samples (10 μL) were mixed in a 1:1 ratio with trypan blue, and the number of cells, the percentage of cells, and the percentage of alive cells were measured over 3 separate trials. No significance was found in the number of hemocytes collected ((**C)** the unpaired two-tailed *t*-test *p* = 0.5772, ns = non-significant, GraphPad Prism 9) or viability between collection methods. ((**D**) alive *p* > 0.05, dead *p* > 0.05; Two-way ANOVA, ns = non-significant, GraphPad Prism 9). (**E**) Hemolymph was extracted from two groups following the methods described in (**a**,**b**); nano-extraction: 10 flies (5 males and 5 females), Centrifugation: 40 flies (20 males and 20 females) with three biological replicates. Samples were loaded into a nanodrop spectrophotometer, protein concentrations were quantified at A280 absorbance with two technical replicates, and this process was repeated over 3 trials. Significant differences in protein concentrations were found between the two collection methods (*** *p* < 0.0001; unpaired two-tailed *t*-test, GraphPad Prism 9).

Hemolymph extraction via centrifugation traditionally requires the use of a large number of flies (20–40 per sample) [16]. Because we had difficulty quantifying centrifuged samples with a lower-than-typical number of flies, we decided to examine how well the nano-extraction method would hold up against using four times the number of flies for centrifugation. We increased the number of flies to 40 for centrifugation, and we were able to obtain a reading on the cell counter comparable to the 10-fly nano-extraction samples, though visually, we noticed a stark difference in the images produced. Centrifuged samples consistently had major clumps of cells and other cellular debris compared to the nano-extraction hemolymph samples, which had a more even distribution of cells (Figure 3A,B).

We revisited hemocyte concentrations and found no significant differences between the two methods despite a fourfold increase in flies for centrifugation (Figure 3C). This is because the cell concentrations from centrifuged samples were widely inconsistent between trials, producing a profound error bar (Figure 3C). This inconsistency could be attributed to the increased occurrence of visible cell clumps produced from spinning down the samples that skew cell counts; yet, trying to resolve this problem by resuspending hemocytes into a more homogenous solution increases the risk of damage to the cells and lowers the rate of viability. Nano-extraction bypasses these implications by producing a more uniform sample, which is consistent in concentrations, making it a more reliable method. However, we were concerned that passing hemocytes through the fine point of the capillary needle may have more of a negative impact on cell viability than the force of centrifugation, so we performed additional hemolymph collections and diluted them with trypan blue to assess the percentage of viable cells retained by both methods. Surprisingly, there was no significant difference in cell viability between the two methods, with both yielding cell survival rates of just under 20% (Figure 3D). Therefore, neither method has superiority over cell viability, but when it comes to generating a consistent uniform sample to be used for further experimentation, nano-extraction has the advantage of reliability over centrifugation while using a fraction of the flies.

Then, we compared the protein concentrations between the 10-fly nano-extraction and 40-fly centrifugation hemolymph samples. The centrifuged samples experienced a significant increase in proteins by a factor of 2.8 compared to nano-extraction (Figure 3E). While the centrifuged samples were able to produce more proteins than the nano-extracted samples, there was increased uncertainty that all the proteins collected were, in fact, hemolymph-derived. The process of spinning down entire flies had an inherent risk of other cells and tissues unintentionally being collected in the supernatant, and this notion was supported by the findings of other cellular debris in both the hemocytometer and cell counter images (Figure 2 and Figure 3A,B). Using a capillary needle to extract the hemolymph with more precision gives increased certainty that the hemolymph is not contaminated with other cellular tissues from the fly that could subsequently skew further experimentation and data analysis.

**Figure 4:**
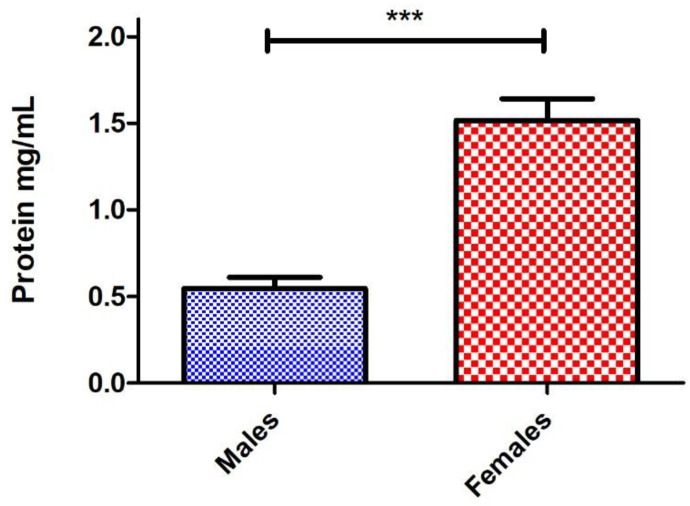
The average concentration of hemolymph collected from individual *D. melanogaster* male and female adult flies. Individual *D. melanogaster* male and female flies were injected with 100 nL of protease inhibitor (PI). The hemolymph was extracted with a nanoinjector, and then the needle was completely emptied into a 0.5 mL microcentrifuge tube containing 2.5 μL of PI before repeating the same process. Samples were loaded into a nanodrop spectrophotometer, and protein concentrations were quantified at A280 absorbance with two technical replicates. An individual trial contained 10 males and 10 females with this process was repeated three times, totaling 30 males and 30 females, and significant differences in protein extracted from males versus females were found (*** *p* < 0.0001; unpaired two-tailed *t*-test, GraphPad Prism 9).

Lastly, we wanted to see if this protocol is capable of extracting sufficient hemolymph from individual flies and identify the ratio of how much hemolymph, on average, is contributed by males compared to females in a unisex sample. We performed nano-extraction from 30 males and 30 females, and protein concentrations were quantified and averaged for each sex. By reducing the final volume of the sample down to 5 μL from 25 μL, we were able to maintain the same protein concentration range of the 10-fly groups (Figure 2C). Naturally, females were found to have a significantly higher protein concentration compared to males, and this could be attributed to their increased body size. However, we discovered that, on average, this occurs by a factor of 3 (Figure 4). This is significant because most experiments typically use a 1:1 ratio (sometimes 3:2) of males to females [17,18] when conducting research to mitigate sex-specific immune effects. Based on our findings, implementing a 3:1 ratio of males to females may have a more balanced contribution by each sex to better neutralize any sex-specific effects.

In summary, this nano-extraction method is capable of consistently generating uniform hemolymph samples with sufficient amounts of viable hemocytes, and the increased certainty of the protein and cellular derivatives collected are of hemolymph origin. Additionally, it facilitates the use of fewer individuals, relieving the burden of having to amplify copious amounts of flies. This method is considered beneficial because it expands the capabilities of using more sensitive fly lines that do not propagate properly in the culture or lines that have rare mutant phenotypes and are produced in low abundance. Likewise, it can increase the number of different experimental groups being investigated simultaneously due to the ability to have lower sample sizes per treatment group.

## 5. Conclusions

Traditionally, centrifugation, as a means for *D. melanogaster* hemolymph collection in adult flies, inconsistently yields variable amounts between experimental replicates. In response, we have developed a novel method of adult fly hemolymph nano-extraction that involves glass capillaries [19]. This method provides the ability to work with experimental groups that have a lower number of individuals. This experimental approach achieves consistent reproducible results with higher amounts of hemocytes and proteins extracted and maintains cell viability compared to centrifugation. Additionally, this method is suitable for single-fly collection as it successfully extracts ample amounts of hemolymph from a single adult fly, allowing for advancements in analyzing infection and immunity processes over the course of a population while also highlighting individual differences. Finally, this method may be applicable to other insects; however, certain aspects of the protocol may need to be adjusted according to the size of the adult or the larval stage of the insect and the amount of hemolymph it contains. For example, we have previously developed another protocol for extracting hemolymph and hemocytes from wild caterpillars [20]. Therefore, it is important to consider that insect hemolymph collection efficiency may depend on the researcher’s technical skills, which makes it particularly challenging to perform a direct comparison of different hemolymphs when isolating protocols among studies conducted in different laboratories and using a variety of insects [21].

## Data Availability

Not applicable.
